# Infection of Primary Bovine Macrophages with *Mycobacterium avium* Subspecies *paratuberculosis* Suppresses Host Cell Apoptosis

**DOI:** 10.3389/fmicb.2012.00215

**Published:** 2012-07-20

**Authors:** Edward Kabara, Paul M. Coussens

**Affiliations:** ^1^Department of Biochemistry, Center for Animal Functional Genomics, Michigan State UniversityEast Lansing, MI, USA; ^2^Department of Animal Science, Center for Animal Functional Genomics, Michigan State UniversityEast Lansing, MI, USA

**Keywords:** apoptosis, efferocytosis, paratuberculosis, programmed cell death

## Abstract

*Mycobacterium avium* subspecies *paratuberculosis* (MAP) is able to survive intracellularly in macrophages by preventing normal phagosome maturation processes utilized to destroy bacteria. Infected macrophages often undergo apoptotic cell death to efficiently present bacterial antigens to the host adaptive immune system in a process known as efferocytosis. Recent studies with *Mycobacterium tuberculosis* (MTB) showed that macrophages infected with MTB are less likely to undergo apoptosis than control, uninfected cells. It is proposed that regulation of macrophage apoptosis is an important immune evasion tactic for MTB. Based on the similarity of MAP and MTB, we hypothesized that MAP-infected macrophages would be resistant to apoptosis compared to uninfected cells within the same culture and to cells from uninfected cultures. Our results demonstrate that, indeed, populations of MAP-infected macrophages contain fewer apoptotic cells than similar populations of control cells, and that MAP infection reduces the sensitivity of infected macrophages to induction of apoptosis by H_2_O_2_. We further demonstrate that MAP-infected cells contain reduced caspase activity for caspases 3/7, 8, and 9. Reduced caspase activity in MAP-infected macrophages is also maintained after H_2_O_2_ induction. This reduction in caspase activity is accompanied by a pronounced reduction in transcription of caspase genes encoding caspases 3, 7, and 8, but not for caspase 9, when compared to control, uninfected cells. Furthermore, MAP infection drastically effects the expression of several host cell proteins important for regulation of apoptosis. Studies using mutant MAP strains demonstrate the importance of bacterial specific factors in the control of host macrophage apoptosis. Together these data demonstrate that MAP specific factors may prevent caspase activity and caspase gene transcription as well as apoptosis signaling protein expression, resulting in decreased spontaneous host cell apoptosis and decreased sensitivity to apoptosis inducing agents.

## Introduction

*Mycobacterium avium* subspecies *paratuberculosis* (MAP), the causative agent of Johne’s disease, is found in over 68% of cattle herds in the United States. The largest percentage of these animals are subclinically infected with the bacterium (USDA–APHIS–VS–CEAH, 2008). Johne’s disease costs the U.S. dairy industry up to $1.5 billion per year in loses (Jones, 1989). A controversial but developing link between MAP and some cases of human Crohn’s disease suggests that MAP may become a significant food safety concern (Spickler, 2006).

One of the key factors that makes MAP such an elusive pathogen is its ability to survive inside host macrophages. Typically, macrophages phagocytose and destroy microorganisms in the host. MAP, however, is able to prevent normal phagosome maturation allowing the bacteria to survive in stalled phagosomes, which become reservoirs for further bacterial growth (Hostetter et al., 2003; Tanaka et al., 2005). To better understand the nature of the host-pathogen interaction in infected macrophages, our group performed a large-scale microarray experiment to study the changes in relative expression of hundreds of host genes in MAP-infected cells. From the genes and pathways found to have altered expression, it was apparent that host cell apoptosis was an important area of focus (Kabara et al., 2010).

Regulation of cell death is extremely important for proper defense against bacteria as well as other intracellular threats to the host. Intracellular bacterial or viral infections should lead to apoptosis of the infected cell to properly destroy the invading pathogen. The apoptotic control of infected cells is a highly conserved mechanism shared by members of both the plant and animal kingdoms (Abramovitch and Martin, 2004; Stuart and Ezekowitz, 2005). After apoptosis of infected cells, macrophages, and other cells then phagocytize the remaining cellular debris and apoptotic blebs containing pathogens, a process called efferocytosis. Efferocytosis then leads to further immune stimulation and clearance of the pathogen from the host. Defects in efferocytosis lead to disease progression in *Francisella novicida* and *Yersinia enterocolitica*, infections as well as in arteriosclerosis (Thorp et al., 2008; Roppenser et al., 2009; Mares et al., 2011). Conversely, if the infected cell dies by necrosis, pathogens will be released and allowed to spread to new targets (Fratazzi et al., 1997; Keane et al., 2000; Sly et al., 2003).

Cell death has recently been a focus of studies in mycobacteria other than MAP, particularly with the human pathogens *Mycobacterium tuberculosis* (MTB) and *Mycobacteria leprae* (ML). MTB can induce apoptosis in heavily infected cells, however, it is now clear that MTB actually suppresses early apoptosis of infected cells as a way for the bacteria to survive intracellularly (Rojas et al., 1997; Keane et al., 2000; Danelishvili et al., 2003; Sly et al., 2003; Lee et al., 2009). ML, in contrast, causes necrosis of infected cells, releasing the bacteria to infect other cells in the host (Chattree et al., 2008).

Recently, Kelly et al. demonstrated that MTB-infected cells induce apoptosis in bystander cells through a cell contact mechanism. The phenomenon described in Kelly et al. (2008) is referred to as “the bystander effect.” These results suggest that when infection rates are low to moderate, studies using whole culture lysates for techniques such as Western blotting, microarrays, and PCR may result in data that does not account for interactions between infected cells and uninfected cells (bystander cells) within the same culture.

The focus of our current study was to understand how MAP may affect apoptosis pathways in bystander and MAP-infected macrophages. We provide evidence that MAP-infected macrophages are less likely to undergo apoptosis than either bystander or uninfected macrophage controls with and without induction by H_2_O_2_. Apoptotic signal transduction in MAP-infected macrophages was also examined. MAP-infected macrophage cultures contained a lower percentage of cells with high caspase activity when compared to bystander and control macrophages, even with strong apoptotic induction. We also observed a reduced abundance of mRNAs encoding several host cell caspases in MAP-infected macrophages. While there were no apparent differences in activation of mitogen activated protein kinase (MAPK) pathway members, we did observe distinct differences in abundance of several key host proteins involved in regulation of apoptosis. Finally, we studied apoptosis of macrophages infected with several MAP mutants to determine if MAP specific factors may be involved in regulation of host cell apoptosis. Our novel results suggest that indeed, MAP genetic elements play a role in preventing apoptosis of infected cells.

## Materials and Methods

### Experimental animals

Uninfected, healthy Holstein cattle at the Michigan State University Dairy Teaching and Research Center were selected as a source of monocyte-derived macrophages (MDM cells) for this study. Cattle were tested for MAP via the IFN-γ test (Bovigam, Biocore Animal Health, Omaha, NE, USA), fecal culture, and ELISA tests (Michigan State University Diagnostic Center for Animal and Population Health, East Lansing, MI, USA). All animal handling procedures were approved by the Michigan State University Committee on Animal Use and Care.

### Cell culture

Isolation of peripheral blood mononuclear cells (PBMCs) was performed using 1× Ammonium Chloride lysing solution on buffy coats extracted from whole blood after centrifugation in a manner similar to previously published experiments (Grone et al., 1999). PBMC were plated in 150 cm^2^ flasks at 1.0–1.5 × 10^8^ per flask and washed with 1× PBS after 24 h to remove non-adherent cells. At 3 days post plating, MDM were harvested using 0.25% Trypsin as suggested by the manufacturer (Invitrogen, Carlsbad, CA, USA). MDM were then replated in RPMI 1640 media supplemented with 10% FBS (complete RPMI) to obtain the necessary number of cells needed for each experiment and cultured as previously described (Kabara et al., 2010).

### Bacterial culture

*Mycobacterium avium* subspecies *paratuberculosis* strain #19698 was purchased from the American Type Culture Collection (ATCC, Manassas, VA, USA) and MAP strain SS149 was generously provided by Dr. S. Sreevatsan (University of Minnesota, Minneapolis, MN, USA). Both MAP strains were grown at 37°C in Middlebrook 7H9 media (Difco Laboratories, Detroit, MI, USA) supplemented with 2 mg/ml Mycobactin J (Allied Monitor, Lenexa, KS, USA) and 10% Middlebrook OADC enrichment (BD Biosciences, Sparks, MD, USA). MAP cultures were grown for 4 months prior to harvesting bacteria for infections. Concentration of MAP was determined via serial dilution and counting on a bacterial hemocytometer. Bacteria were stored at 4°C prior to infection of MDM cells as previously described (Kabara et al., 2010). Periodic acid-fast tests were performed to check each culture was a pure mycobacteria culture.

### Bacterial staining and infection protocol

*Mycobacterium avium* subspecies *paratuberculosis* cultures were vortexed for 1 min to disrupt clumps prior to use as previously described (Kabara et al., 2010). Bacteria were labeled to allow subsequent differentiation of infected and bystander cells via flow cytometry. Based on the initial bacterial concentration, a premeasured volume of bacterial culture was placed in a 1.5-ml eppendorf tube. An equal volume of 1,1′-dilinoleyl-3,3,3′,3′-tetramethylindocarbocyanine perchlorate (DiI; Invitrogen, Carlsbad, CA, USA) was added to stain infecting bacteria prior to infection and the bacteria/staining solution was vortexed and placed in a dark area for 30 min. After this time, the stained bacteria were centrifuged at 13,000 × *g* for 5 min and washed three times with 1 ml 1× PBS. Post washing, labeled bacteria were suspended in PBS. A volume of the labeled bacteria solution was added to each MDM-MAP infection plate such that the total amount of labeled bacteria would be 20 times the amount of MDM present in each sample (MOI = 20). MAP-infected cultures were washed with 1× PBS at 24 h post infection to remove all non-phagocytosed bacteria as previously described (Kabara et al., 2010).

### Apoptosis labeling

**Figure 1:**
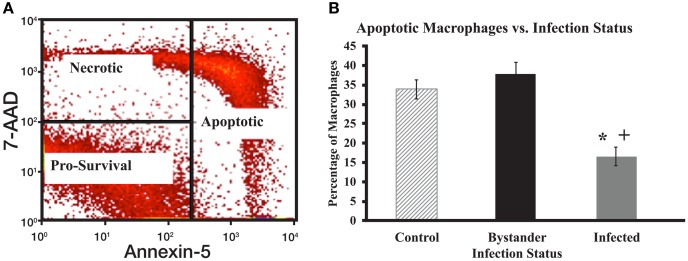
**Apoptotic gating strategy and levels of spontaneous apoptosis in cultured macrophage populations**. **(A)** Cells were initially gated after flow cytometry to focus on live macrophage cells and then according to 7-AAD and Annexin V staining intensity. Cells with strong staining for Annexin-5 were considered early or late apoptotic. For analysis purposes, data from both populations were combined to examine all apoptotic cells [Right rectangle in **(A)**]. Cells with strong 7-AAD staining, but little Annexin-5 staining were considered necrotic (upper left quadrant). Cells with little staining for either 7-AAD or Annexin V were considered pro-survival (lower left quadrant). **(B)** The percentage of total apoptotic macrophages was determined by flow cytometry as described for **(A)**. The bars represent mean results obtained from MDM cultures isolated from six individual healthy Holstein cattle. Error bars represent Standard Error of the Mean (SEM) between the six biological replicates. A “+” indicates significantly different from control, uninfected cells at *p* < 0.05 and a “*” indicates significantly different from the bystander macrophage populations at *p* < 0.05.

Monocyte-derived macrophages cells were harvested in 1× Annexin V binding buffer (BD Biosciences, Franklin lakes, NJ, USA). Annexin V-FITC and 7-AAD were then added to the cell suspension to stain for apoptotic and necrotic cells, respectively. This cell suspension was then placed in a dark area at room temperature for 15 min. Post labeling, 1× binding buffer was added to the cell solution to quench the staining reaction. Stained cells were then analyzed using a BD FACSCalibur flow cytometer. Cells were electronically gated based on size and granularity to remove debris from final analyses. Cells were then separated into infected and bystander populations, based on fluorescence (or lack of fluorescence) from the DiI-labeled MAP. After initial gating, cells were divided into one of three groups: pro-survival, apoptotic, and necrotic. Cells that showed low staining for both Annexin V and 7-AAD were considered pro-survival cells. Cells that show low Annexin V staining, but stain with 7-AAD are considered necrotic cells. Cells with high Annexin V staining regardless of 7-AAD status are considered apoptotic cells (Figure 1A).

### Caspase activity assays

Monocyte-derived macrophages cells were labeled using the CaspaTag system for monitoring activity of caspases 3/7, 8, and 9, essentially as recommended by the manufacture (Millipore, Billerica, MA, USA). This label binds to the active form of each of target caspases. Caspase activity was analyzed using a BD FACSCalibur flow cytometer. As before, cells were initially gated based on size and granularity, then by MAP infection status. Cells within these groups were further divided into two additional groups: those cells showing low caspase activity and those cells showing high caspase activity. All cells contain some caspase activity and this methodology is a standard way of distinguishing apoptotic cells (high caspase activity) from non-apoptotic cells (low caspase activity) as described by the manufacturer. To test the division of cells into these two groups based on caspase activity, cells were treated with hydrogen peroxide (H_2_O_2_), which is known to stimulate caspase activity (Jones et al., 2000). We observed a clear shift of cells moving from the low caspase activity group to the high caspase activity group, confirming the use of these categories.

### Antibody labeling

Sister cultures of MDM were treated with macrophage-colony stimulating factor (M-CSF; 100 ng/μl for 5 min after 18 h serum deprivation) or granulocyte M-CSF (GM-CSF; 100 ng/ml for 24 h in our previously described complete RMPI media) prior to harvesting samples (Santa Cruz Biotechnology, Santa Cruz, CA, USA). MDM were harvested in 1× Fixation/Permeabilization buffer (eBioscience, San Diego, CA, USA) prior to intracellular labeling. MDM were labeled using antibodies specific to several host proteins involved in the MAPK pathway or in apoptosis (Table 1). Antibody labeling occurred while in 1× Fixation/Permeabilization buffer and all cells were washed using 1× Fixation wash solution. Antibody labeled MDMs were diluted in 300 μL of 1× PBS. Protein concentration and phosphorylation was evaluated in labeled cells using a BD FACSCalibur flow cytometer as per the manufacturer’s instructions.

**Table 1:** **Antibodies used in flow cytometry**.

Antibody	Company	Stock number	Dilution
IgG1 Anti-FLIP	Enzo Life Sciences	ALX-804-428	1:25
IgG1 Anti-FADD	Abcam	AB10519	1:50
IgG1 Anti-BAD	Abcam	AB62480-100	1:50
IgG1 Anti-p-AKT	Santa Cruz Biotechnology	SC-81433	1:25
IgG1 Anti-p-BAD	Santa Cruz Biotechnology	SC-271963	1:25
IgG1 Anti-MCL-1	Abcam	AB31948	1:25
IgG1 Anti-ERK	Sigma-Aldrich	M3807	1:50
IgG1 Anti-pERK	Sigma-Aldrich	M8159	1:50
IgG1 Anti-pJNK	Sigma-Aldrich	J4750	1:50
IgG2A Anti-pp38	Sigma-Aldrich	M8177	1:50
IgG2A Anti-JNK	Sigma-Aldrich	SAB4200176	1:25
IgG2A Anti-TRADD	AbD Serotec	MCA4825Z	1:50
IgG2B Anti-p38	Sigma-Aldrich	M8432	1:25
IgG2B Anti-AKT	GenWay Bio	20-787-276352	1:25
Alexa Flour 488 Anti-IgG1	Invitrogen	A21121	1:1000
Alexa Flour 488 Anti-IgG2A	Invitrogen	A21131	1:1000
Alexa Flour 488 Anti-IgG2B	Invitrogen	M32501	1:200

*As part of the MAPK flow cytometry study, several different antibodies were used. For each antibody, the Company, Stock Number, and dilution are listed above*.

### Quantitative real time reverse transcriptase PCR

Control, uninfected MDM or MDM cells infected with MAP strain SS149 at a MOI 20:1 were lysed and processed to obtain total RNA using the 5 Prime RNA extraction kit as suggested by the manufacturer (5 Prime, Gaithersburg, MD, USA). Quantitative Real Time reverse transcriptase PCR (qRT-PCR) primer design, cDNA synthesis, PCR methods, and data analysis were performed as previously described (Kabara et al., 2010). A full list of primers and sequences are available on the Center for Animal Functional Genomics website (http:/www.cafg.msu.edu).

### Statistical analysis

After data collection, all biological replicates for each sample were combined to develop a mean response. Error measurements for graphs were made via the standard error of the mean method. Outliers were removed via Interquartile Range determination. Samples were analyzed as a randomized block design using a mixed model procedure (PROC MIXED; SAS inst., Cary, NC, USA). To estimate sampling day effect on our measures the following model was used:

$$Y_{ij}=\mu +S_{i}+B_{j}+e_{ij}$$

Where *Y_ij_* is the dependent variable for cow_I_ in sample*_j_*, μ is the overall mean, *S_i_* is the fixed effect of treatment (Control, Bystander or MAP-Positive), *B_j_* is the random block of animal and *e_ij_* is the residual. Comparisons of treatment Least squares mean were calculated and adjusted using the Tukey–Kramer method. Treatments were considered significantly different at *p*-value < 0.05.

## Results

### Spontaneous apoptosis in MAP-infected MDM cells

There were significant differences in the percentage of cells undergoing spontaneous apoptosis between populations of MAP-infected, bystander, and control macrophages. Uninfected sister cultures of MDM were collected from each cow to serve as control macrophages, while MAP-infected and bystander macrophages in MAP-infected MDM cultures were separated based on bacterial fluorescence as described above. MAP-infected macrophages had a significantly lower relative percentage of apoptotic cells when compared to both bystander and control macrophages (*p* < 0.0001 for each sample; Figure 1B). Populations of bystander macrophages tended to contain a higher percentage of apoptotic cells than control cells from uninfected cultures, however this difference was not significant (*p* = 0.33). No significant differences were apparent when the relative percentage of necrotic cells was examined in the three populations (data not shown). As expected, populations of MAP-infected macrophages showed a larger percentage of pro-survival cells when compared to bystander and control cells (*p* < 0.0001 for each sample, data not shown). Bystander cell populations tended to display fewer pro-survival macrophages than control cells, but his difference was also not significant (*p* = 0.33). This leads us to conclude that MAP-infected macrophages are less likely to spontaneously enter apoptosis than cells from control, uninfected cultures, or bystander cells. Bystander cells were not significantly different than cells from control uninfected cultures.

### MAP-infected macrophages are more resistant to induction of apoptosis then control or bystander macrophages

**Figure 2:**
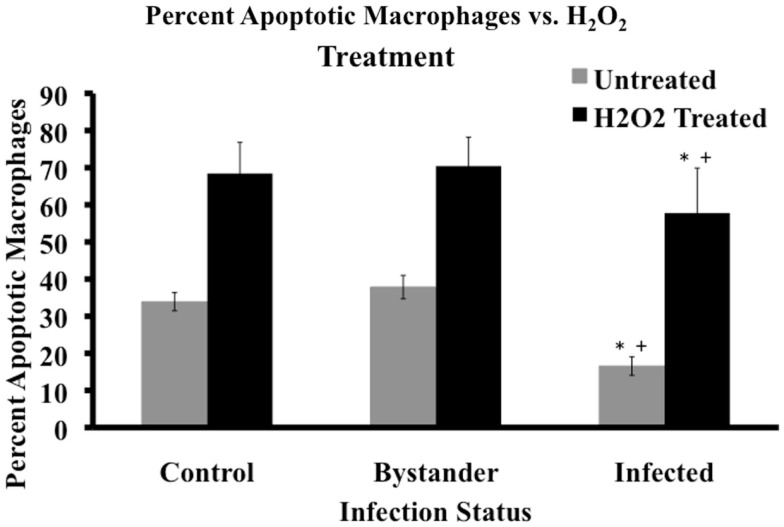
**Cell status post apoptotic induction**. Percentage of apoptotic macrophages in populations of MAP-infected, bystander, and uninfected control cells was determined by flow cytometry after 20 min of 100 μM H_2_O_2_ treatment. Bars represent the average results of MDM cultured from six healthy Holstein cattle. Gray bars represent the percentage of apoptotic cells pre-treatment, while black bars represent the percentage of apoptotic macrophages after treatment in the three cell groups. Error bars represent Standard Error of the mean (SEM) between the six biological replicates. A ^+^indicates significantly different from control, uninfected cells at *p* < 0.05 and *indicates significantly different from bystander macrophages at *p* < 0.05.

Based on differences in spontaneous apoptosis observed between MAP-infected, bystander, and control cell populations, we studied the relative resistance of these three cell groups to induction of apoptosis. Hydrogen peroxide (H_2_O_2_) is a well-known apoptotic inducing agent (Ryter et al., 2007). We chose to use 100 μM H_2_O_2_ for 20 min, based on time course and dose-response curve studies with uninfected MDM (data not shown). All cell populations exposed to H_2_O_2_ displayed a higher percentage of apoptotic macrophages than their untreated sister cultures (*p* < 0.05). When H_2_O_2_-treated macrophages were compared across the infection groups, H_2_O_2_-treated MAP-infected MDM cell populations contained a significantly lower relative percentage of apoptotic cells than either bystander or control macrophage populations (*p* = 0.0132 and 0.0064, respectively; Figure 2). No other significant differences were observed between the three groups (data not shown).

### MAP-infected macrophages have much less caspase activity then control or bystander macrophages

*Mycobacterium avium* subspecies *paratuberculosis*-infected macrophage populations had a lower percentage of spontaneously apoptotic cells than control or bystander macrophage populations. MAP-infected macrophages also showed a higher resistance to H_2_O_2_ driven apoptosis induction. Given the well-known importance of caspases in apoptosis, we determined if MAP infection had an effect on caspase activity (Ulukaya et al., 2011). We focused on monitoring activity for caspases 3/7, 8, and 9 as these are central to the apoptotic cascade (Ulukaya et al., 2011). Because H_2_O_2_ efficiently induced apoptosis in all cell types, we chose this reagent to induce the cells during caspase studies.

**Figure 3:**
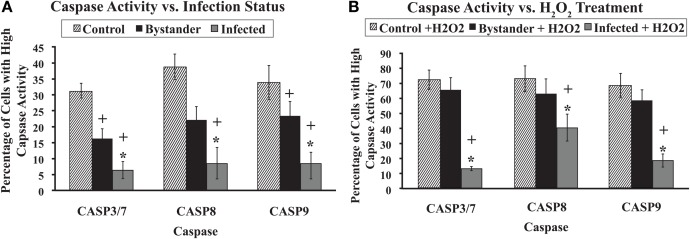
**Caspase 3/7, 8, and 9 activity with and without H_2_O_2_ treatment**. The percentage of cells with high activity for caspases 3/7, 8, and 9 in various cell populations was determined using flow cytometry, as described in Materials and Methods. Bars represent the average results of MDM cultured from six healthy Holstein cattle. Error bars represent Standard error of the mean (SEM) between the six biological replicates. A ^+^indicates significantly different from control, uninfected cell cultures at *p* < 0.05 and *indicates significantly different from bystander macrophage populations at *p* < 0.05. Data in **(A)** represents the percentage of cells with high caspase activity without any apoptosis induction. **(B)** Shows the percentage of cells that display high caspase activity after 20 min of exposure to 100 μm H_2_O_2_.

Caspases 3/7, 8, and 9 all showed very similar patterns of activity in untreated cells. Uninfected, control macrophage cultures contained the highest relative percent of cells with high caspase activity. Bystander macrophage populations contained an intermediate percentage of cells with high caspase activity. MAP-infected macrophage populations contained the lowest percentage of cells with high caspase activity. Regardless of the caspase studied, all three of these groups were significantly different from one another (*p* < 0.05; Figure 3A).

As before, cells were exposed to 100 μM H_2_O_2_ for 20 min to induce apoptosis. The relative percentage of cells with high caspase activity in control, bystander, and MAP-infected MDM populations was significantly higher following H_2_O_2_ induction compared to populations from untreated sister cultures, regardless of the caspase studied (*p* < 0.05). MAP-infected macrophage populations contained a significantly lower relative percentage of cells with high caspase activity than either control or bystander MDM populations for caspases 3/7, 8, and 9 (*p* < 0.05). However, no significant differences were observed between control and bystander macrophage populations for any of the caspases studied (Figure 3B).

### MAP infection reduces caspase mRNA abundance

**Figure 4:**
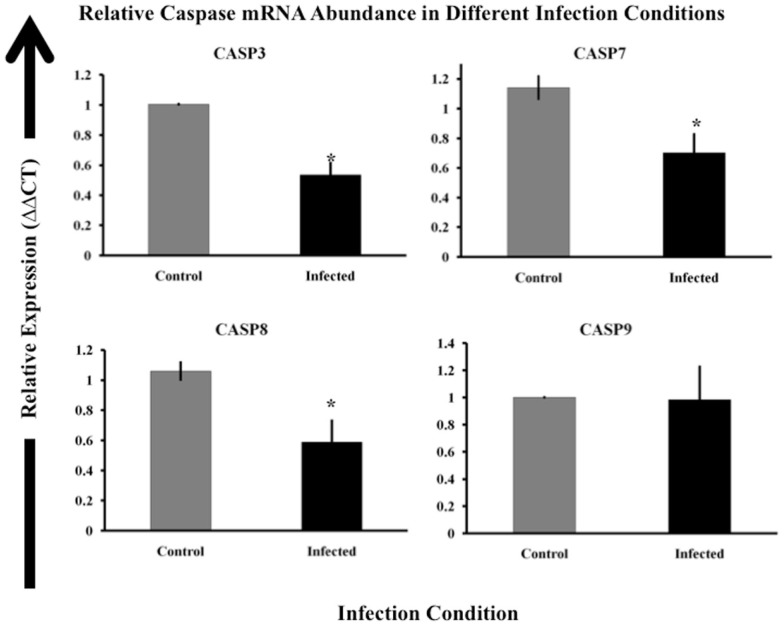
**Relative expression of caspase genes in control and MAP-infected macrophages**. Abundance of mRNA encoding caspases 3, 7, 8, and 9 was determined via RT-qPCR as described in Materials and Methods. The ΔΔCt method was used to determine relative mRNA abundance using beta-actin as the control gene (Livak and Schmittgen, 2001). The uninfected sample (control) is indicated by gray boxes while infected samples are shown as black boxes. Bars represent the average results of MDM cultured from eight healthy Holstein cattle. Error bars represent standard error of the mean (SEM) between the eight biological replicates. Samples marked with a star (*) indicate samples that are significantly different then control samples at *p* < 0.05.

Based on our finding that MAP-infected cell populations had generally lower caspase activity, we next focused on caspase mRNA abundance. We studied the relative abundance of mRNA encoding caspases 3, 7, 8, and 9. Differences in caspase gene expression or mRNA abundance could explain the observed reduction in caspase activity in MAP-infected macrophages. For this work, we used the bovine MAP strain SS149. SS149 routinely infected over 85% of cells in a culture at a MOI of 20. We infected MDM with DiI-labeled SS149 and determined the percentage of macrophages that were infected in each culture using flow cytometry. We considered macrophage cultures infected with MAP at or over 85% to be heavily infected and thus suitable for whole culture analyses. We observed significantly less mRNA encoding caspases 3, 7, and 8 in macrophages infected with MAP SS149 than in control, uninfected macrophages (*p* < 0.05). However, no significant differences were observed for mRNA encoding caspase 9 (Figure 4). Thus, reduced expression of caspase mRNA in MAP-infected macrophage cultures could, at least partially, explain the observed reduction in caspase 3/7 and 8 activity. However, other factors would need to be considered to explain the observed reduction in caspase 9 activity.

### MAP-infected and control macrophages have distinct differences in apoptotic protein expression

**Figure 5:**
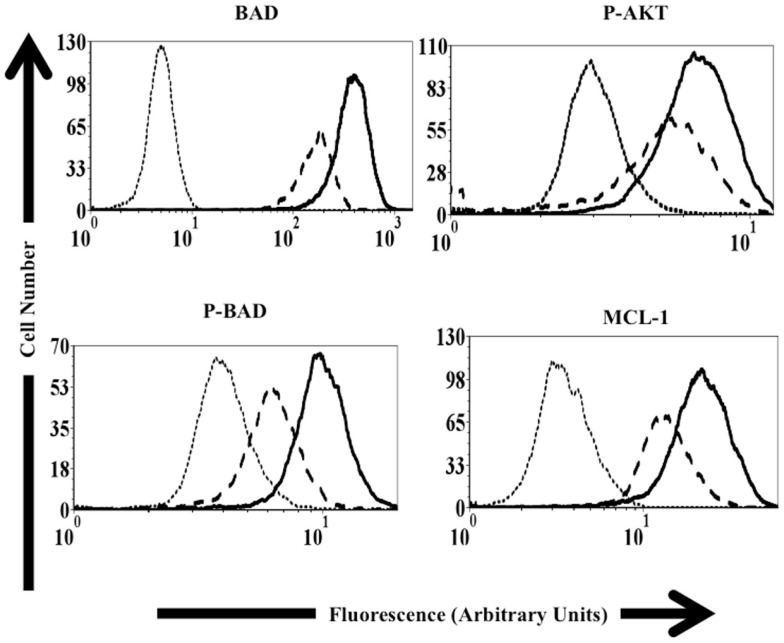
**Protein expression in control and MAP-infected macrophages**. Representative histograms used to determine relative protein expression via flow cytometry as described in Materials and Methods. The dotted line represents isotype control samples (Samples exposed to only the secondary antibody). The dashed line represents data from MAP-infected macrophage populations. Solid black lines represent control, uninfected macrophage samples. Samples used to study p-AKT, BAD, and p-BAD were exposed to M-CSF prior to analysis. Samples used to study MCL-1 were exposed to GM-CSF prior to analysis.

As caspase 9 mRNA levels were not altered in macrophage cultures following MAP infection, we sought other mechanisms to account for observed differences in caspase 9 activity in MAP-infected cells. Work from other groups suggested that host gene or protein expression of BAD, AKT, and MCL-1 displayed significant differences in control cultures and macrophage cultures infected with either MAP, ML, or MTB (Maiti et al., 2001; Hasan et al., 2006; Kabara et al., 2010). To determine if MAP-infected macrophages showed differences in protein expression in our system, MDM were infected with MAP SS149 for 24-h and treated with either M-CSF to stimulate production/activation of BAD and AKT or GM-CSF to stimulate activation of MCL-1. After M-CSF treatment, control uninfected MDM exhibited significantly more unphosphorylated and phosphorylated BAD than untreated cells. M-CSF treatment of MDM previously infected with MAP SS149 also enhanced levels of unphosphorylated and phosphorylated BAD, but not to the extent observed in uninfected cells (Figure 5). A similar reduction in the phosphorylation of AKT was also observed in MAP-infected cultures as compared to uninfected cultures (Figure 5). In each case observed differences were significant (*p* < 0.05). MAP-infected MDM treated with GM-CSF demonstrated significantly lower expression of MCL-1 when compared to control uninfected cultures also treated with GM-CSF (*p* < 0.05; Figure 5). In each case, there were also significantly fewer cells positive for the various proteins in MAP-infected MDM cultures than in control uninfected cultures (*p* < 0.05).

### Mutant MAP strains fail to control host cell apoptosis

**Figure 6:**
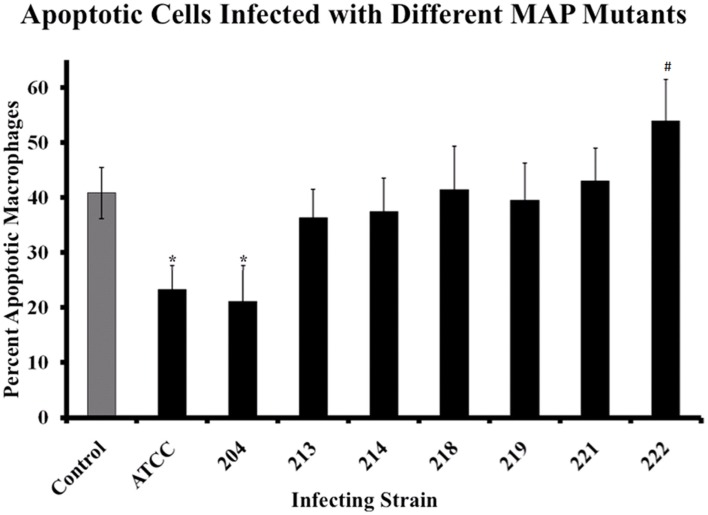
**Macrophages infected with MAP mutants fail to prevent host cell apoptosis**. The percentage of apoptotic macrophages was determined as described in Materials and Methods. Bars represent the average results of MDM cultured from four healthy Holstein cattle. Error bars represent standard error of the mean (SEM) between the four biological replicates. An ^#^indicates significantly different from ATCC#19698 (ATCC)-infected macrophages at *p* < 0.05 while a *indicates significant difference from control, uninfected macrophages at *p* < 0.05. The nature of each mutation is presented in Table 2, as described in the text.

As we examined the host response to MAP, we were also interested in what MAP factors might be involved in regulating host cell apoptosis. To examine the role that specific genes play and identify areas of the MAP genome involved in alteration of macrophage apoptosis, several mutant MAP strains were obtained from the Johne’s Disease Integrated Program (JDIP) in a blind study. For this work, we were only interested in the relative percent of cells undergoing spontaneous apoptosis following MAP infection, compared to control uninfected cells, thus data from bystander macrophages is not shown. In Figure 6, the percentage of spontaneous apoptotic cells is shown for control (uninfected), ATCC 19698-infected (wild-type), and several strains of mutant MAP-infected macrophages. As before, ATCC 19698-infected macrophage cultures contain a lower relative percentage of apoptotic cells than control, uninfected cultures (*p* < 0.05). One MAP mutant (204) appeared to also significantly reduce macrophage apoptosis whereas most others did not. One mutant (222) actually enhanced apoptosis in infected cultures, relative to ATCC infected cells (*p* < 0.05). Details on the individual strains used as well as the mutant gene in each of these MAP strains is shown in Table 2. Our data indicates that MAP1872c mutated in strain 222 is likely important for MAP infection driven regulation of host cell death.

**Table 2:** **Mutant MAP strains**.

Strain number	Mutation	Mutated gene
204	Phage/site directed mutation	*lsr2*/deletion (MAP0460)
213	Transposon mutagenesis (Tn5367)	MAP1566
214	ppiA gene (MAP0011)	MAP0011
218	Homologous recombination	MAP4287c
219	Transposon mutagenesis (Tn 5367)	MAP2408c
221	Homologous recombination	MAP1719c
222	Transposon mutagenesis (Tn 5367)	MAP1872c

*Several different mutant MAP strains were used in our study. For each mutant MAP strain used in our study, the nature of the mutation as well as the gene involved is shown*.

## Discussion

Apoptosis of infected cells is an important immune control tactic in defense against intracellular pathogens (Fratazzi et al., 1997; Keane et al., 2000; Sly et al., 2003). However, some pathogens may prevent host cell apoptosis, circumventing efferocytosis, and ensuring limited immune system detection (Pena et al., 2009). A report by Kelly et al. (2008) showed control of host cell apoptosis exerted by MTB in both infected and bystander cells (uninfected cells in an infected culture). While this report clarified the regulation of apoptosis in MTB-infected macrophages, comparatively little is known about how MAP might regulate apoptosis in Johne’s disease. In this study we sought to determine if MAP altered host cell apoptosis, to investigate the bystander effect during MAP infection, and to begin examining bacterial factors that may control apoptosis in MAP-infected macrophages. Our results clearly demonstrate that MAP-infected macrophage cultures contain a higher percentage of pro-survival cells when compared to bystander and control cell populations. Thus, our results with MAP suggest a similar control over host cell apoptosis as described for MTB (Kelly et al., 2008). Unlike MTB, however, we did not observe a significantly enhanced level of apoptosis in bystander cell populations compared to control, uninfected cultures.

As apoptosis is heavily regulated in the host, we next wanted to study how MAP-infected, bystander, and control cells reacted to induction of apoptosis by an exogenous agent. MAP-infected macrophages were significantly less sensitive to induction of apoptosis by H_2_O_2_ when compared to both control and bystander cells, though H_2_O_2_ did enhance apoptosis in the infected population relative to similar untreated cells (Figure 3). This data demonstrates that MAP infection reduces the ability of only MAP-infected macrophages to enter apoptosis as no protection was extended to bystander macrophages in an infected culture. This indicates that the MAP infection driven mechanism of macrophage survival likely relies on the presence of bacteria within the cell.

Since MAP-infected macrophages are less likely to undergo apoptosis than control or bystander macrophages, any efforts by the host to induce apoptosis specifically in MAP-infected macrophages would most likely induce apoptosis in surrounding cells and tissues, while leaving the intracellular bacteria relatively unscathed. An important consequence of a lower relative percentage of apoptotic cells in MAP-infected macrophages would be lack of efferocytosis. Therefore, less MAP antigen would be presented to the adaptive immune system, which could have a deleterious effect on development of a proper immune response.

To elucidate pathways involved in the reduction of apoptosis in MAP-infected macrophages, we studied the caspase activity of cells under different infection conditions. MAP-infected macrophage populations had the lowest percentage cells with high caspase activity, even with strong apoptotic induction by H_2_O_2_ (Figure 3). Since caspases are central to apoptotic pathways, we conclude that this block may be an important part of MAP-mediated regulation of host cell apoptosis. A reduction in caspase activity could readily explain the significant differences in apoptosis observed in our study, as less caspase would be available to signal within MAP-infected cells. Although there was an increase in caspase activity following treatment in the infected macrophage population, this was significantly less pronounced than in either uninfected control cultures or in the bystander cell population. These data suggest that infection of cells with MAP blocks the apoptosis pathway at a point upstream of caspase activation.

Danelishvili et al. (2010) studied the role MTB plays in host caspase activity. Two MTB proteins Rv3654c and Rv3655c were identified that bound host proteins involved in transcriptional regulation of caspases. They demonstrated increased caspase 8 activity in macrophages infected with an MTB Rv3654c mutant when compared to wild-type MTB. While Rv3654c does not have a homolog in MAP, sequences exist that may be homologous to Rv3655c (444491-444262 in the MAP genome; *E*-value < 10^−20^). Based on our caspase activation data, the results of Danelishvili et al. and the homology between MAP and MTB, we studied the relative abundance of several caspase mRNAs in MAP-infected macrophages. By selecting only cultures with little to no bystander macrophages present, we were able to marginalize the effect bystander cells would have on the results of a whole culture based analysis, such as qRT-PCR. We demonstrated that MAP infection reduces the mRNA abundance for genes encoding caspase 3, caspase 7, and caspase 8 when compared to uninfected controls (Figure 4). If reduction of caspase 3, caspase 7, and caspase 8 mRNA abundance manifest as a reduction in caspase 3, caspase 7, and caspase 8 protein, then less caspase would be available to signal in the cells offering a possible explanation for reduced caspase 3/7 and caspase 8 activity. However, because we observed no similar reduction in caspase 9 mRNA abundance this explanation does not appear to extend to the observed reduction in caspase 9 activity in infected macrophages.

In addition to our work studying the caspase and apoptosis cascade in MAP-infected cells, we also studied other pathways that may be altered by MAP infection and that may account for the observed loss of caspase 9 activity. We found significantly lower expression of BAD, phosphorylated BAD (p-BAD), phosphorylated AKT (p-AKT), and MCL-1 in MAP-infected cultures relative to control, uninfected cultures (Figure 5). AKT phosphorylation is an important step in preventing apoptosis and reduced levels of p-BAD would also tend to favor apoptosis (Danial, 2008). Thus, reduced p-BAD and reduced p-AKT could both lead to a reduction in overall apoptotic potential in cells. Reduced levels of MCL-1, a well-known anti-apoptosis signaling protein, would tend to favor apoptosis in MAP-infected macrophages (Thomas et al., 0000). However, this effect is obviously offset by some other, perhaps as yet unknown, balance within MAP-infected macrophages. Signals from BAD, AKT, and MCL-1 eventually converge at the mitochondria, which is extremely important in regulation of apoptosis in MTB-infected cells (Duan et al., 2002; Gan et al., 2005; Cadieux et al., 2011). It is possible that pro-apoptotic signals (i.e., reduced MCL-1) may be present upstream of the mitochondria, but MAP prevents loss of mitochondrial membrane integrity. This in turn would prevent cytochrome C release and apoptotic signal transduction via caspase 9. Further work must be done to clarify the proteins and/or pathways involved in MAP induced changes in regulation of host cell apoptosis via caspase 9.

In addition to the aforementioned proteins, our group also studied the expression of several other host proteins in MAP-infected macrophages. MAPK signal transduction was previously studied by our group, but we did not specifically study the MAP-infected, bystander, and control macrophages during our initial work (Sommer et al., 2009). However, consistent with our previous results (Sommer et al., 2009), we observed no significant differences in protein expression or activation within this group of proteins (p38, ERK1/2, and Jun/SAPK). Furthermore, we examined expression of several other apoptosis signaling proteins, including TRADD, FADD, and FLIP in MAP-infected and control cells. Again, we observed no significant differences between expression of these proteins in MAP-infected and control cells.

Bystander macrophage populations tended to be similar to uninfected control cell cultures in spontaneous apoptosis. However, we observed significantly fewer cells with high caspase activity in bystander cell populations when compared to control, uninfected cells. When bystander macrophage populations were treated with H_2_O_2_, no differences were observed between the control or bystander macrophages in terms of apoptosis or caspase activity. These data suggest that caspase signaling systems in bystander cells are reduced relative to those in control uninfected cells, but these systems can be induced to initiate apoptosis following induction by external stimuli. Mycobacteria are known to secrete several proteins and lipids into culture media, as well as exchange lipids with infected macrophages (Av-Gay and Everett, 2000; Vergne et al., 2004; Danelishvili et al., 2010; Cadieux et al., 2011). Based on this information, bystander macrophages in MAP-infected cultures may be exposed to low levels of mycobacterial products that reduce caspase activity, but do not disable the system as in the infected cells. These results distinguish MAP-infected macrophages from MTB-infected macrophages, where a significant bystander effect was observed (Kelly et al., 2008).

Finally, we studied apoptosis in macrophages infected with several MAP mutants and compared the relative percentage of apoptotic host macrophages that found in uninfected control cultures and to populations of cells infected with MAP strain ATCC #19698 (Figure 6). Host macrophage populations infected with MAP mutant strain 222 contain a higher percentage of apoptotic cells compared to populations of cells infected with ATCC #19698. MAP 222 contains a mutation in the mbth_2 gene, encoding an iron acquisition protein (Zhu et al., 2008). Iron acquisition is extremely important in intracellular bacterial survival and perhaps even more so in MAP. As little is known about mbth_2 and MAP iron metabolism in general at this time, more work is needed to fully understand what role this protein may plays in host cell death regulation and/or bacterial survival in macrophages.

The first major impact of this work is that future studies investigating cell death pathways, as well as other mechanisms, should consider bystander macrophages in MAP-infected cultures. Previous work by our group and others studying MAP-macrophage interactions have studied a whole infected culture as a single unit (Bermudez et al., 1999; Kabara et al., 2010). As demonstrated above, MAP-infected macrophages display a lower relative percentage of apoptotic cells than bystander macrophages. Therefore, use of whole culture based methods, such as qRT-PCR and western blotting, without accounting for potentially opposing features of infected and bystander cells, may yield results related to the *average* response of a population as opposed to the actual response of individual cells to infection. This would also be true of studies employing such techniques on infected tissues. Apoptosis gene expression results from our previous microarray work were inconclusive with regard to MAP infection either up-regulating or down-regulating apoptosis, likely a direct result of the bystander effect (Kabara et al., 2010). Pro-survival transcripts in MAP-infected macrophages were likely being mixed with pro-apoptotic transcripts in bystander macrophages and muting the true effects of MAP on infected cells. Consequently, precautions should be taken to monitor the relative percent of cells in a culture that are actually infected.

The second major impact of this research is the role that MAP infection and apoptosis regulation play in treatment of the disease. Previously, we suggested that MAP infection prevents apoptosis and efferocytosis as a way to circumvent the adaptive immune response. Based on this hypothesis, mycobacterial specific factors may play a very important role in the altered regulation of host cell death, such as the role Rv3654c plays in MTB regulation of host cell apoptosis (Danelishvili et al., 2010). Future studies considering the role MAP prevention of host cell apoptosis plays in the whole immune system may better explain the spread of this disease in the host and the external environment.

## Conclusion

Apoptosis of MAP-infected macrophages is important for the effective clearance of the bacterium from the host. However, our data demonstrates that MAP-infected macrophage populations contain a lower percentage of spontaneously apoptotic cells than uninfected cell populations in the same culture. Furthermore, these cells are much less likely to undergo apoptosis even after strong induction from agents such as H_2_O_2_. MAP-infected macrophages also show a drastically lower ability to activate caspases and contain lower caspase 3, 7, and 8 mRNA levels, which could be an explanation for the reduction in the ability of infected cells to enter apoptosis, relative to bystander macrophages, and cells from uninfected control cultures. Future work focused on the bacteria driven host apoptotic regulation may result in new treatments and vaccine candidates for Johne’s disease.

## Conflict of Interest Statement

The authors declare that the research was conducted in the absence of any commercial or financial relationships that could be construed as a potential conflict of interest.
